# Work Precarity, the Great Recession, and Disparities in HbA1c Changes in Older Workers

**DOI:** 10.1093/geroni/igaf122.3686

**Published:** 2025-12-31

**Authors:** Miriam Mutambudzi

**Affiliations:** Syracuse University, Syracuse, New York, United States

## Abstract

Racial health disparities are exacerbated during economic recessions, when employment disruptions including precarious employment intensify. This study examines whether cumulative precarity, occurring before and during the Great Recession, contributes to worsening diabetes risk, as measured by HbA1c, among older Black and White workers. Data from the Health and Retirement Study (2006–2016) were used to examine changes in HbA1c over 4-year period among 1,573 older Black and White workers. Precarity i.e. experiencing involuntary unemployment, short job tenure, or reduced work hours, and was classified as pre-recession only (2006), Great Recession only (2008–2010), both periods, or neither (referent). Conditional change regression models assessed 4-year follow-up biomarker changes from baseline, adjusting for relevant covariates. Baseline HbA1c strongly predicted follow-up HbA1c (β = 0.75, p < 0.001). While precarity did not show a uniform effect across all participants, race-specific analyses revealed meaningful differences: race*precarity interactions indicated stronger effects among Black participants for pre-recession (β = 0.39, p = 0.028), Great Recession (β = 0.37, p = 0.003), and both periods (β = 0.37, p = 0.005). Stratified analyses confirmed that among Black participants Great Recession only precarity predicted higher follow-up HbA1c (β = 0.30, p = 0.031), with pre-recession only and both-period exposure showing similar marginal trends (p = 0.056). Among White participants, no significant associations were observed. Employment precarity during and prior to the Great Recession was associated with higher follow-up HbA1c among older Black workers, but not Whites, highlighting racial disparities in vulnerability to economic shocks.

